# Gut-microbiome profiles among Soil-transmitted helminths (STHs) infected Ethiopian children enrolled in the school-based mass deworming program

**DOI:** 10.1371/journal.pntd.0012485

**Published:** 2024-10-15

**Authors:** Bineyam Taye, Zeleke Mekonnen, Kenneth D. Belanger, Emily R. Davenport

**Affiliations:** 1 Department of Biology, Colgate University, Hamilton, New York, United States of America; 2 Institute of Health, School of Medical Laboratory Sciences, Jimma University, Jimma, Ethiopia; 3 Department of Biology, Huck Institutes of the Life Sciences, Institute for Computational and Data Sciences, Pennsylvania State University, University Park, Pennsylvania, United States of America; Consejo Nacional de Investigaciones Cientificas y Tecnicas, Fundación Mundo Sano, ARGENTINA

## Abstract

**Background:**

Soil-transmitted helminths (STHs) and mutualistic gut microbes coexist in the gastrointestinal tract. However, limited data exist regarding how STH infections are associated with gut microbiome profiles.

**Method:**

We conducted a cross-sectional analysis of baseline data collected in a longitudinal study to identify and explain differences in microbial communities between STH-infected and non-infected Ethiopian school children. We collected 138 stool samples and analyzed them for STH infection using standard direct wet mount and Kato Katz methods. The gut microbiome profiles were characterized using targeted amplicon sequencing of the 16S rRNA gene from the total DNA extracted from the stools.

**Results:**

Children infected with *Trichuris trichiura* showed significantly lower microbial diversity than those who were non-infected (p<0.05). We also observed significant difference in microbiome composition based on *Trichuris trichiura* infection status (PERMANOVA p< 0.01). A comparison of microbial taxa at the genus level among participants infected with different helminth species showed a significant increase in *Agathobacter* relative abundance among children infected with *Trichuris trichiura* compared to non-infected subjects (adjusted p = 0.001).

**Conclusions:**

Our results indicate that changes in the gut microbiome composition may vary depending on the species of helminth present. Further studies should investigate how *Trichuris trichiura* selectively alters microbiome composition compared to other STH species.

## Author summary

The coexistence of soil-transmitted helminths (STHs) and mutualistic gut microbes in the gastrointestinal tract is poorly understood. We studied the gut microbiome in Ethiopian school children infected and non-infected with soil-transmitted helminths (STHs). We found that children infected with *Trichuris trichiura* showed significantly lower microbial diversity. Comparing microbial taxa at the genus level, we observed a significant increase in *Agathobacter* relative abundance among children infected with *Trichuris trichiura* compared to non-infected subjects. These findings indicate that the gut microbiome composition may vary depending on the species of helminth present. Further research is needed to understand how *Trichuris trichiura* selectively alters the gut microbiome compared to other STH species. Data on soil-transmitted helminths (STHs) and their relationship to the microbiome would be valuable for planning future public health interventions. This is particularly important for controlling helminth infections while considering the microbial community.

## Introduction

Soil-transmitted helminthiases are worm infections from roundworm (*Ascaris lumbricoides*), whipworm (*Trichuris trichiura*) and hookworm (*Ancylostoma duodenale* and *Necator americanus*), among others worm species [1]. STH infections affect one third of the world’s population and can cause debilitating disease, but are also sometimes present without causing recognizable symptoms in their host [2]. The World Health Organization (WHO) suggests controlling infections caused by STHs in high-risk countries by implementing mass deworming programs that use potent antihelmintic drugs without considering the infection status [3]. This intervention effectively reduces the prevalence and intensity of STH infections [4]. It also helps mitigate the adverse health effects of these infections [5]. However, this program does not consider the potential impact of removing STH parasites on other gut microbes that coexist in the same environment. STH parasites and gut microbes have evolved together in the gastrointestinal niche and are believed to have adapted to influence each other in complex ways [6–8]. One hypothesis being explored by researchers is that helminth infection could lead to systemic immune responses, directly impacting the community of gut microbiota [9,10] or indirectly affecting immune responses through interactions with gut microbiota [11]. Others have suggested that the presence of antimicrobial peptides (AMPs) in helminth excretory/secretory products (ESPs) could have both lethal and non-lethal effects, altering the composition of gut microbiota [12,13].

Research on non-human mammals has revealed that helminth infection correlates with changes in the gut microbiome, which usually leads to an increase in microbial diversity [14,15]. Results of limited, small studies in humans on the impact of STHs on microbiome composition have varied, with some suggesting that the presence of some STHs does not significantly alter the gut microbiome [16,17] and others indicating that STH infection results in changes in microbial diversity within the gut [17–20]. Similarly, the dynamics of specific microbial clades of interest (such as the common gut bacteria Clostridiales, Enterobacteria, and Firmicutes) in relation to STH infection or treatment show mixed results across studies [21–23].

Most microbes that reside in the gut are not harmful, and many are beneficial. They help the host digest food, support the development of the immune system, modulate essential behaviors, and even impact heart disease, among other potential effects [24]. Additionally, they compete with harmful organisms to establish a foothold within the host [25]. Thus, it is important to examine what factors may lead to changes in the gut microbiome and how they interact with other organisms that share the same environment. Consequently, investigating interactions between helminths and the gut microbiome is crucial for determining how the microbiome changes due to soil-transmitted helminth (STH) infection. STHs may decrease the populations of non-STH pathogens by cross-immunity or competitive inhibition [23], potentially disrupting the development of the normal microbial community and increasing the risk of dysbiosis[10]. This effect might be more significant in countries where helminth infections are common, as children in these areas are typically infected with helminths at a young age before their gut microbiome has stabilized [26]. Therefore, understanding the relationship between helminths and the microbiota could provide valuable insights for planning future public health interventions to control helminth infections while taking the microbial community into consideration.

Currently, there is a significant shortage of data that investigates the impact of STH infection on the microbiomes of children participating in mass deworming programs–a particularly important and vulnerable subset of the population. Therefore, this study utilized baseline data to describe the profiles of gut microbial communities in relation to STH infection and anthelmintic treatment history among schoolchildren in Ethiopia.

## Methods

### Ethics statement

The study was approved by the Institutional Review Boards (IRBs) of Colgate University, Hamilton, NY, USA (IRB Reference number: FR-S20-03/20) and Jimma University Institute of Health, Ethiopia (IRB Reference number: IHRPGD/786/20). We obtained written or fingerprint consent from parents or legal guardians after informing them of the study procedures and aims. Confidential numerical identifiers are assigned to each child to ensure participant privacy, and all participant information remains password-protected in electronic files. Children were also informed about their ability to withdraw from this study at any time without jeopardizing their right to receive any services in the school. Children who were found to have intestinal parasites were subsequently treated with standard anti-parasitic drugs as per local treatment guidelines. The children were also informed about their ability to withdraw from this study without jeopardizing their right to receive any services at their school.

### Study setting and context

The detailed study area map has been published elsewhere [27]. Briefly, data was collected in Jimma Town, Ethiopia. Jimma is 352 km southwest of Addis Ababa and at 7040’21.94” N and 36050’12.12” E with an elevation/altitude of 1713.59 meters above sea level. Jimma has a tropical wet and dry climate with an annual mean temperature of 15.28°C, 91.72 millimeters of annual precipitation, and 74.29% humidity. The projected total population of Jimma Town was estimated at 207,573 in 2021/22, based on data from the 2007 census (120,960) [28]. The study was conducted in an area that covered 14 public elementary schools managed by the Jimma Town Office of Education. Out of these schools, ten primary schools were selected based on their geographical distribution in the town. The primary schools included in the study were Jiren, Ginjo, Tulema Keneni, Abdi Gudina, Jimma, Mendera, Seto Yido, Dilfire, Kito, Hirmata, and Hamle-19. Jimma zone was specifically chosen because soil-transmitted helminths are common in this population, and these schools participated in mass deworming programs. In Jimma Zone, mass deworming of all school-aged children with single doses of anthelmintics (400 mg albendazole or 500 mg mebendazole) biannually was adopted [29]. The study area has a low level of sanitation because many households do not have access to clean drinking water.

### Study design

This study is part of a longitudinal research project to investigate the gut microbiome of Ethiopian school children before and after being treated with mass deworming drugs. Two rounds of fecal specimens were collected, one at the beginning of the study (baseline, April to May 2020) before drug administration and a second 12 months after treatment. The portion of the study described here examines only the samples collected pre-deworming. Existing records and new survey information were used to identify basic health characteristics of the children, including their state of treatment for STHs, dietary habits, living conditions, and sanitary habits, to provide information about conditions that may affect intestinal microbes. The analysis reported here is a cross-sectional study of only the baseline data collected before treatment with anthelmintics to identify and characterize differences in microbial communities in STH-infected versus non-infected Ethiopian school children.

### Study population

The original cohort enrolled 1036 study subjects from 10 schools and was described in detail elsewhere [30]. For the current analysis, we used a random sampling method to include 140 participants. After excluding insufficient samples, 138 subjects were selected for the final analysis. We ran a sensitivity analysis to compare the demographic and lifestyle characteristics information between subjects included and excluded for microbiome analysis and found no significant difference **(S1 Table).**

### Measurement and data collection

#### Sociodemographic and selected lifestyle

A questionnaire was used to collect information about various sociodemographic and lifestyle factors related to children. The questionnaire was translated into local languages like Oromiffa and Amharic to ensure the accuracy of the responses. The collected data included details about age, gender, place of residence, family size, parent education, and occupation, as well as anthropometric measurements such as height and weight. In addition, the questionnaire also captured information on certain clinical and lifestyle variables like deworming history, vaccination history, anthelminthic drug use, and frequency of eating vegetables, fruits, roots and tubers, meat, eggs, cereals, legumes, milk, and tea or coffee **(S2 File).**

#### Fecal sample collection and parasitological analyses

Fecal samples were collected in a leak-proof plastic container and divided into two portions. The first fecal sample was used for parasitological examination and processed immediately for microscopic examination of helminths by Kato Katz techniques at Jimma University Institute of Health Neglected Tropical Disease Laboratory. The other fecal sample was collected and immediately transferred to Norgen Stool Nucleic Acid Collection and Preservation Tubes (Norgen Biotek Corp), then sent to Jimma University and frozen within 2 hours of collection. These tubes were transported to Colgate University in the USA and stored at -80°C until DNA extraction.

#### Microscopical examination of soil-transmitted helminthiases

Stool samples were analyzed for STH infections using Kato-Katz techniques on the same collection day [31]. The Kato-Katz thick smears (Sterlitech, USA) were conducted in duplicate following the World Health Organization’s protocol [31,32]. A child was considered to have a soil-transmitted helminth (STH) infection if at least one of their fecal samples tested positive for one or more species of STHs. Fecal egg counts were measured per-species for fecal samples that tested positive for STH by calculating the geometric mean number of eggs per gram of feces (EPG) in each sample. This method helps to account for outliers that may occur due to a few study subjects having very high egg counts [33].

#### DNA extraction, library preparation, and sequencing

One aliquot of each frozen fecal sample was thawed prior to extract DNA extraction. DNA was extracted using the DNeasy PowerSoil Pro kit (Qiagen, Germany) according to manufacturer’s instructions using up to 250 mg of fecal sample per extraction. Homogenization was performed using two 30-second pulses on a Beadbeater (BioSpec, Inc. OK, USA) at 4°C. The concentration and purity of isolated DNA were measured using the NanoDrop One (ThermoFisher Scientific, MA, USA). The integrity of genomic DNA was tested by resolving DNA extracts on a 0.8% agarose gel by electrophoresis followed by visualization with ethidium bromide staining. Microbial DNA extraction was confirmed by PCR amplification of the V4 variable region of 16S rDNA using primers 515F-Y and 806R according to Earth Microbiome Project (EMP) protocols (earthmicrobiome.org/protocols-and-standards/16s). After initial sample extraction, aliquoted DNA samples were stored at − 80°C and later shipped to MR DNA molecular laboratory (MR DNA, TX, USA) for sequencing on the Illumina MiSeq platform following standard Illumina protocol. The sequence raw dataset in FASTQ file type was demultiplexed using FASTQ Splitter 64 bit v19.07.10.

#### Amplicon sequencing reads processing

Sequence reads were subjected to quality assessment using FastQC [34]. Raw sequence data were quality filtered using Trimmomatic V.39 to remove low-quality sequences (quality score < 20) and reads shorter than 50 base pairs. ASVs were then generated using DADA2 version 1.26 [35] and R 4.2.2 [36] following the basic methodology outlined in the ‘DADA2 Pipeline Tutorial (1.10). Briefly, adapter sequences were removed using the Cutadapt software [37]. Identical reads were then dereplicated, and a sample inference algorithm was applied. Filtered high-quality sequences were aligned and classified taxonomically using the SILVA (SILVAv138) [38] reference database after merging paired reads and removing chimeras. Contaminating archaeal, mitochondrial, and chloroplast sequences were removed. Relative abundance was calculated on the filtered dataset.

### Statistical analysis

Data were analyzed using phyloseq (version 1.26.1) [39] in R (v4.2.2). We normalized the data by rarefying to 20,000 read sampling depth without replacement before the diversity analysis Alpha diversity was calculated using the Chao1 [40] and Shannon indexes [41] to examine the association between helminth infection and fecal microflora diversity. To determine the similarity of bacterial communities between helminth-infected and non-infected study groups, we calculated beta-diversity analysis using weighted UniFrac [42] and Bray-Curtis dissimilarity [43] indices using the microbiome package [44], ‘vegan’[45] and pairwiseAdonis (v0.4) in R (v4.2.2) [46]. A pairwise permutational multivariate analysis of variance (PERMANOVA) was then used to determine if the differences in the weighted UniFrac and Bray-Curtis indices were statistically significant in helminth-infected versus non-infected samples. Additionally, we ran unconstrained ordination using principal component analysis (PCA) based on Aitchison distances at the genus level [47]. For differential abundance analysis between helminths infected versus non-infected, analysis of compositions of microbiomes was carried out with bias correction (ANCOM-BC) [48].

This tool estimates the logarithmic fold difference in the abundance of features based on a variable of interest and provides statistical significance for the difference [49]. The Benjamini–Hochberg method was applied for multiple comparisons. Adjusted P values ≤ 0.05 were considered statistically significant. Comparisons of α-diversity by helminth infection status were assessed using the Wilcoxon rank-sum test. Lastly, an algorithm for discriminating high-dimensional bacterial biomarkers features, linear discriminant analysis (LDA) effect size (LEfSe) [50], was used to determine significant differences in bacterial taxonomies (LDA score > 2.5). A post-hoc power analysis using G*Power software version 3.1.9.2 [51] revealed that a total sample size of 138 (18 Trichuris positive vs. 120 negatives) offered 80% power to detect significant differences in α (Shannon index) diversity and at least two-fold differences in bacterial taxa abundance using a two-tailed t-test at a significance level of p = 0.05.

## Results

### Characteristics of the study subjects

**Table 1** shows the distribution of socio-demographic characteristics of our study cohort. Of the 138 schoolchildren enrolled in the study, 58.7% were females, and the majority (74.6%) were urban dwellers. The mean age of children was 10.9 years (SD 2.5 years), ranging from 6 to 17 years. 58.7% of children had Bacillus Calmette-Guerin (BCG) vaccination, and (50%) had a history of deworming treatment in the past six months, while 64.5% had a deworming treatment in the past year. **Table 2** provides the distribution of intestinal parasites. Overall, 46.4% (64/138) of the children were infected with at least one intestinal parasite, and 39.1% (54/138) were infected with any soil-transmitted helminths (STHs; *Ascaris lumbricoides*, *Trichuris trichiura*, *Schistosoma mansoni*, hookworm, or others). *Ascaris lumbricoides* was the most prevalent helminth (31.2%), followed by *Trichuris trichiura* (13.0%).

**Table 1:** Distribution of sociodemographic characteristics.

Variables		Frequency	Percentage
Sex			
	Male	57	41.3
	Female	81	58.7
Age (years)			
	5–8	29	21.0
	9–12	72	52.2
	>12	37	26.8
Place of residence			
	Urban	103	74.6
	Suburb/Rural	35	25.4
Weight in Kg			
	17–29	58	29
	30–34	56	39.9
	>43	24	31.2
			
Deworming in past year			
	Yes	89	64.5
	No	49	35.5
Deworming in past six months			
	Yes	69	50
	No	69	50
Mode of Delivery			
Vaginal	Yes	126	91.3
C-Section	No	12	8.7
Bacillus Calmette-Guerin (BCG) vaccination*			
	Yes	81	58.7
	No	57	41.3

**Table 2:** Distribution of Helminths parasite infections in the study subject.

Individual parasite infections		Frequency	Percentage
	*Ascaris lumbricoides*	43	31.2
	*Tricuris trichiura*	18	13.0
	*Schistosoma mansoni*	11	8.0
	Hookworm	1	0.7
	Others*	4	2.9
Any parasite infection**			
	Yes	64	46.4
	No	74	53.6
Any STHs infection***			
	Yes	54	39.1
	No	84	60.9

• Others include Hymenolepis spp. Or Taenia spp

**Any parasite was defined as being positive for at least one parasite (including other none STHs)

***Any Soil-Transmitted Helminths defined positive for Ascaris lumbricoides, Trichuris trichiura, or Hookworms

### Overview of sequencing output

To investigate the overall bacterial composition of the gut microbiome, we profiled the taxonomic content of fecal samples using a 16S rRNA gene sequencing approach (see methods). A total of 34,825,073 quality-filtered sequences were obtained across all samples, with an average of 252,355 sequences per sample. A total of 1,731 unique amplicon sequence variants (ASVs) were generated, and after filtering to remove low-prevalence ASVs (<10% of samples), 881 ASVs were retained for the final analysis. Rarefaction analysis showed that the number of new ASVs tended to be stable as sequencing depth increased past ~50k reads per sample, indicating that the acquired sequencing depth of all samples faithfully captures the microbial diversity within samples **(S1 and S2 Figs).**

### Microbial community diversity among helminth-infected and non-infected schoolchildren

**Fig 1:**
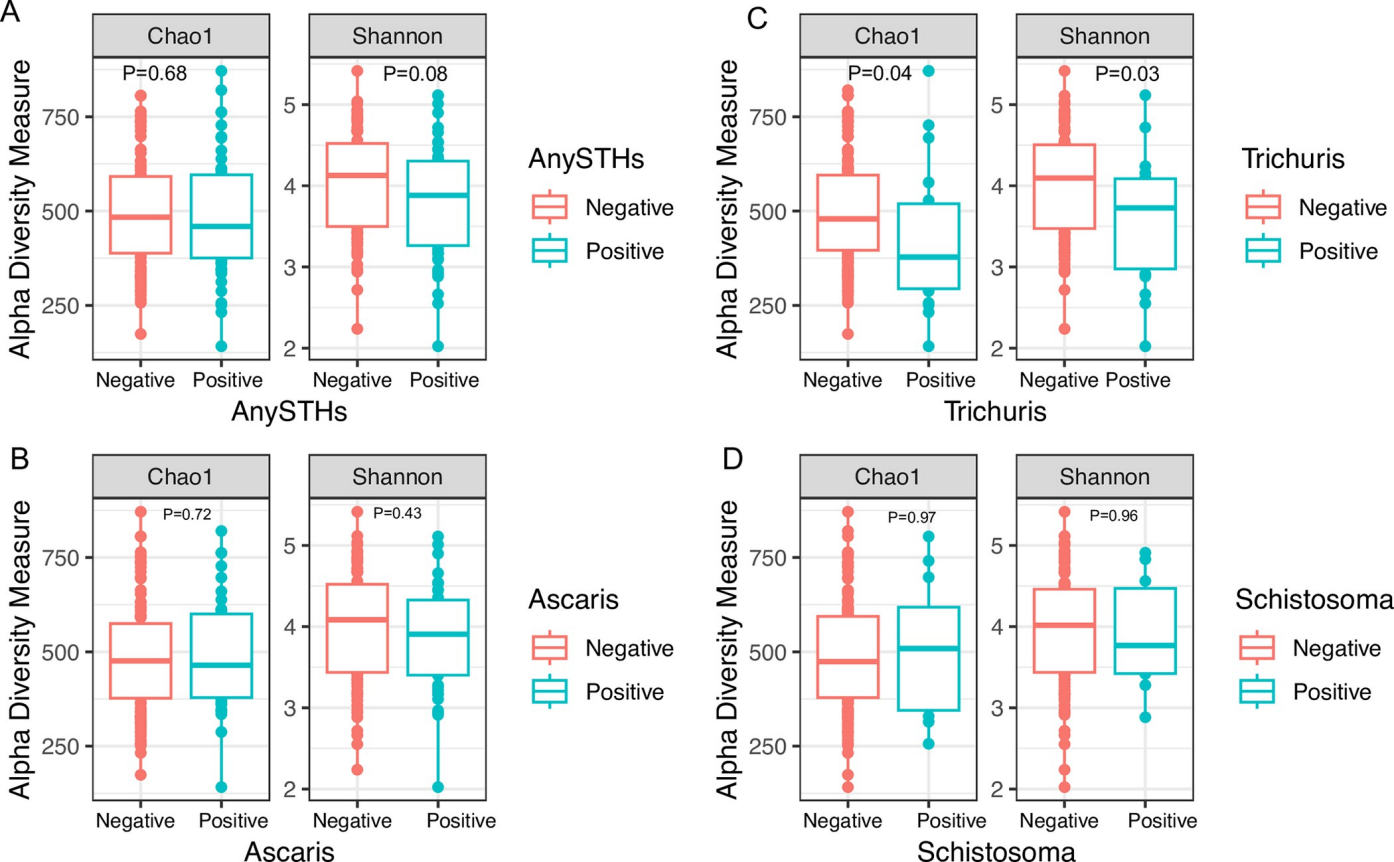
Microbiota diversity by helminth categories. Boxplots show alpha diversity (either Chao1 or Shannon diversity) as a function of helminth infection status. A) Any STHs positive vs. Negative, (B) Ascaris positive vs. Negative, (C) Trichuris positive vs Negative, (D) Schistosoma positive vs. Negative. Statistical significance was assessed with the Wilcoxon rank sum test. ns = not significant and ** P<005.

**Fig 2:**
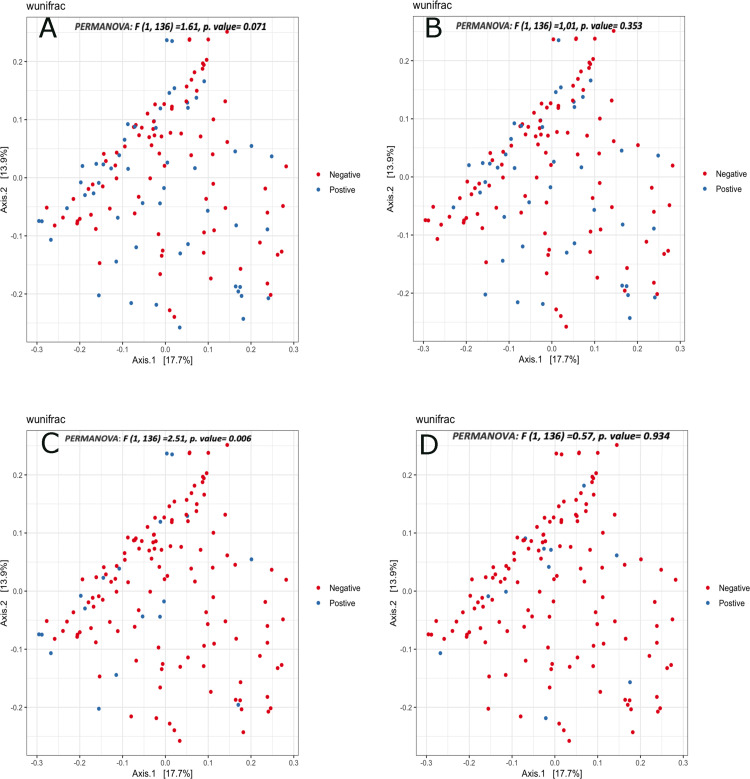
Principal coordinate analysis (PCoA) based on weighted UniFrac matrix similarity of overall fecal microbiota composition by different Helminth infection status. (A) AnySTHs positive vs. Negative, (C) Trichuris positive vs. Negative, (B) Ascaris positive vs. Negative, (D) Schistosoma positive vs. Negative. P-values resulted from PERMANOVA analysis between compared groups.

First, we aimed to assess whether helminth infection was associated with within sample diversity differences in our cohort. Overall, Shannon and Chao 1 diversity indices demonstrated inconsistent trends of microbial diversity found among helminth infected vs. uninfected subjects (**Fig 1A–1D**). Interestingly, children infected with *Trichuris trichiura* showed significantly lower microbial diversity (P<0.05**, Fig 1C**), unlike our observations for other helminth species. We further used the weighted UniFrac distance metric to evaluate microbiome compositional similarity by helminth infection status **(Fig 2).** We found that for the most part there are not broad compositional differences in the microbiome depending on infection status, but the exception again being with *Trichuris* (PERMANOVA P < 0.01). We also ran other beta diversity metrics including Bray Curtis dissimilarity to investigate the compositional dissimilarities between helminths infected and non-infected samples and found no statistically significant difference between helminth infection categories **(S3 Fig).** Similarly, unconstrained ordination by means of principal components analysis (PCA) indicated no clustering of samples from the helminths infected or non-infected. **(S4 Fig).** Further analysis was conducted to examine the potential relationship between specific demographic and lifestyle factors (BCG vaccination, mode of delivery, history of diarrhea) and microbial diversity. The results showed no significant variations in alpha and beta diversity indices (**S5 and S6 Figs).**

### Gut microbial taxonomic differences among helminth-infected and non-infected schoolchildren

**Fig 3:**
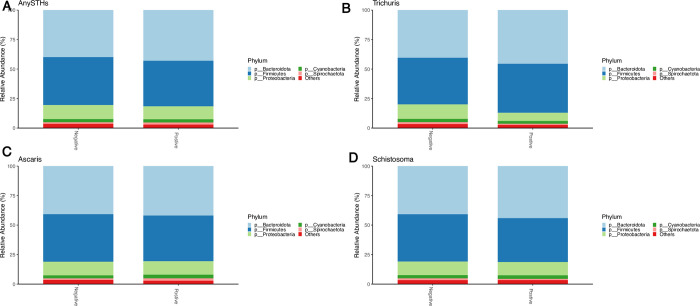
Relative abundances of microbiota at the phylum level by different Helminths infection status. (A) AnySTHs positive vs. Negative, (C) Ascaris positive vs. Negative, (B) Trichuris positive vs. Negative, (D) Schistosoma positive vs. Negative. The ‘Other’ group pools together phyla with very small prevalence.

To further examine potential differences in the gut microbiome composition of STH-infected and non-infected children, we compared the relative abundance of various taxa in the two groups. At the phylum level, the predominant taxa were Bacteroidetes, Firmicutes, and Proteobacteria in the gut bacterial community of both study groups **(Fig 3).** We ran ANCOM-BC tests to determine if individual bacterial taxa differ relative abundance between helminth-infected and non-infected subjects. None of the helminth species showed significant differences in microbial relative abundances between infected and non-infected when correction for multiple testing was applied.

**Fig 4:**
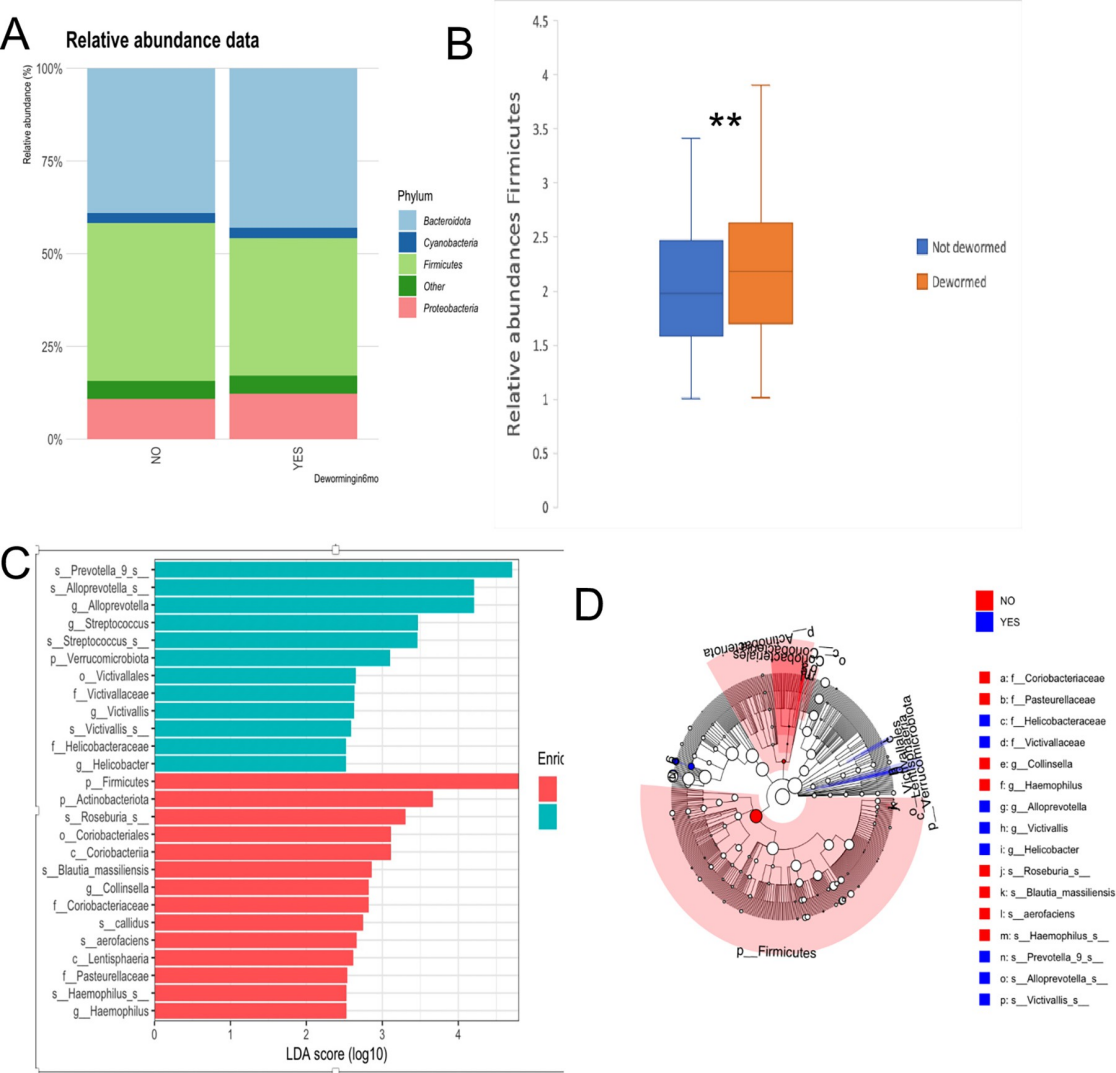
Relative abundances of microbiota at the phylum level (A) and (B) Box plot independent t-test log relative abundances of Firmicutes. (C) Taxonomic cladogram and bar chart (D) obtained from linear discriminant effect size (LEfSe) analysis highlights specific bacterial taxa that were relatively more abundant in the deworming group (red) or more abundant in non-deworming (blue). Significant bacterial taxa were determined by the Kruskal-Wallis test (P, 0.05) with an LDA score greater than or equal to 2.5. ** P<0.05.

To assess the influence of the history of anthelmintic drugs as part of school deworming programs on the relative abundance of individual bacterial taxa, we performed a comparison of phyla abundances between dewormed students and those who did not receive an anthelmintic treatment (Fig 4). The distribution of phyla differs between the two groups (Fig 4A), and a significantly higher Firmicutes abundance was observed in children who received deworming treatment in the past year compared to non-dewormed (adjusted *p* < 0.05, **Fig 4B).** This higher level of Firmicutes in dewormed subjects was confirmed using linear discriminant effect size (LEfSe) analysis [50] (Fig 4D), as were variations of additional taxa at the class, order, family, genus, and species levels (Fig 4C and 4D).

**Fig 5:**
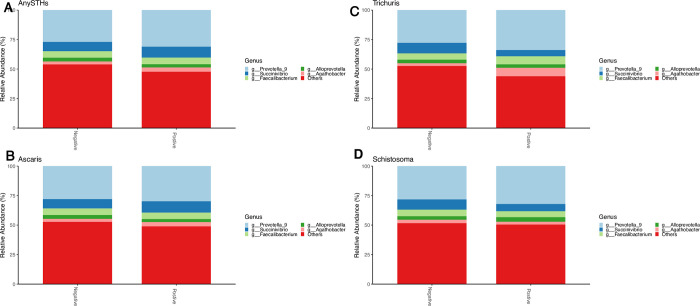
Relative abundances of microbiota genus level by different parasite infection status. (A) AnySTHs positive vs. Negative, (B) Ascaris positive vs. Negative, (C) Trichuris positive vs. Negative, (D) Schistosoma positive vs. Negative.

**Fig 6:**
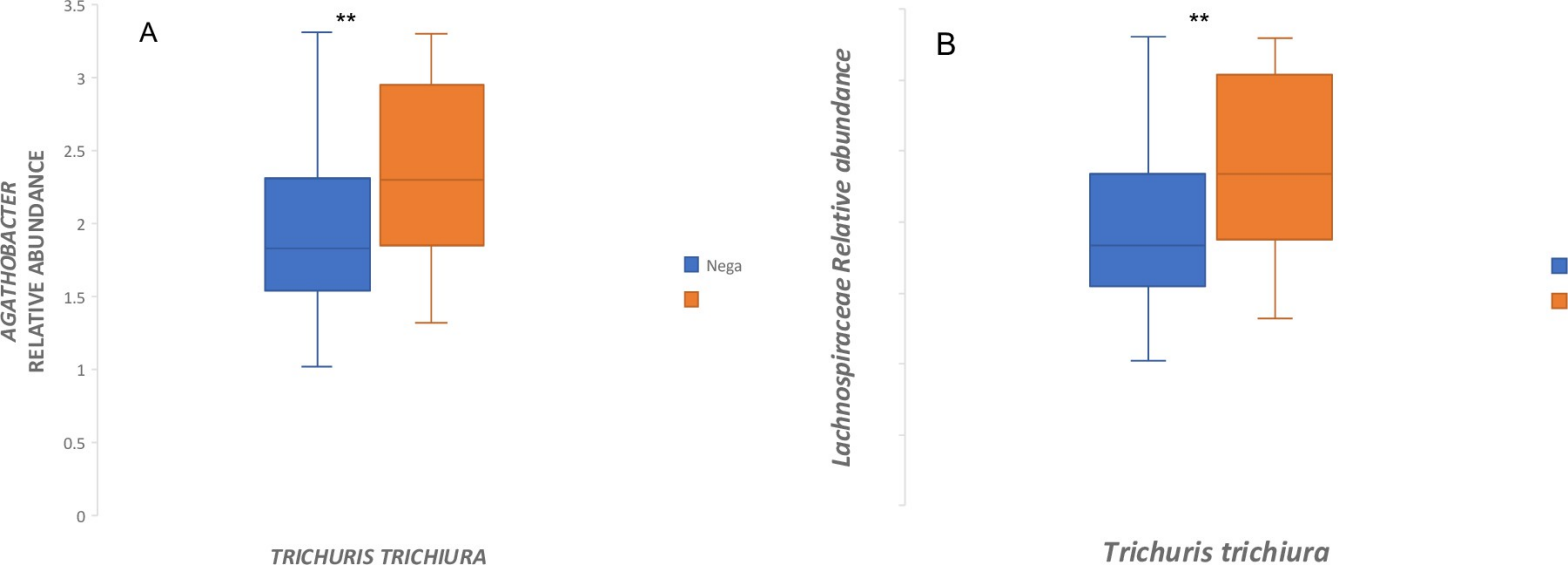
Relative abundances of microbiota at the genus and family level by Trichuris infection status. (A) Independent t-test of log relative abundances of *Agathobacter* between Trichuris positive vs. Negative. (B) Independent t-test of log relative abundances of Lachnospiraceae between Trichuris positive vs. Negative. **P<0.05.

**Fig 7:**
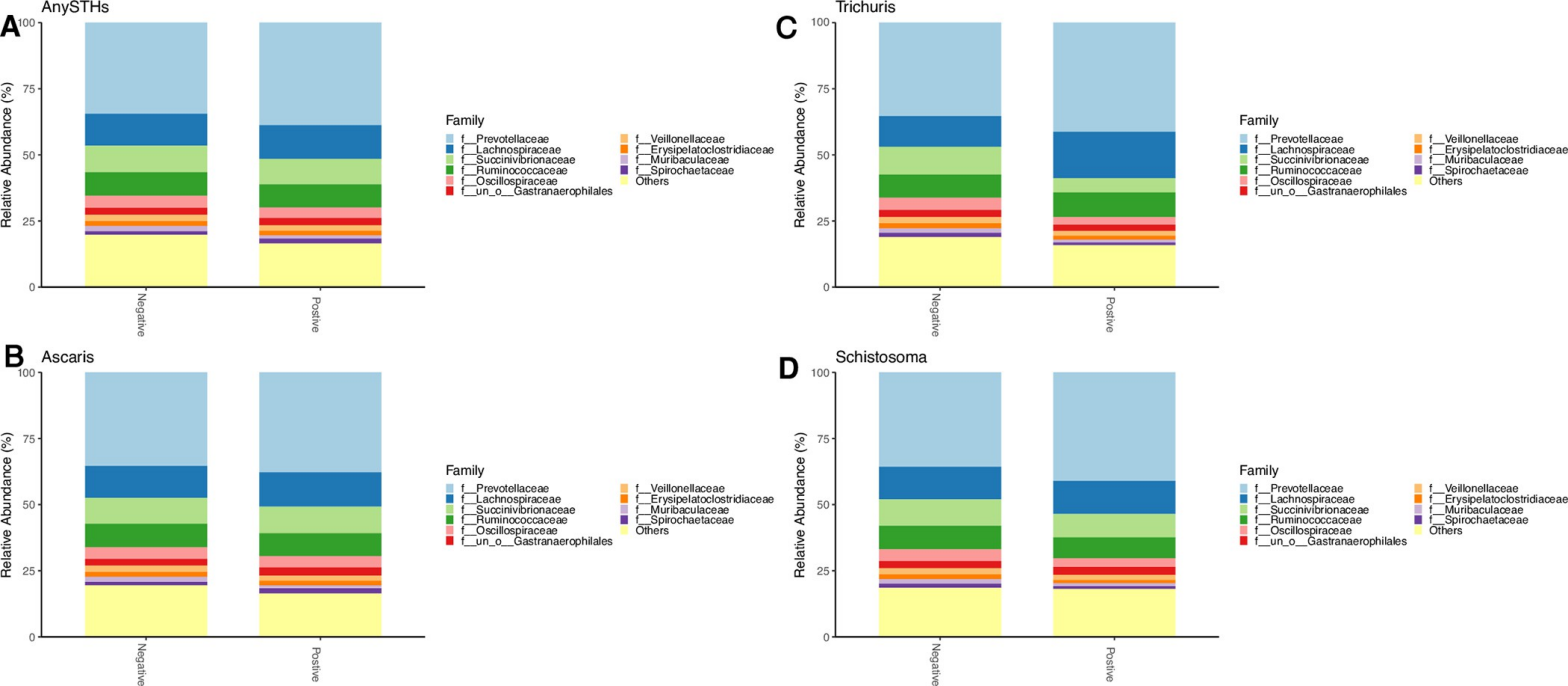
Relative abundances of microbiota at the family level by different Helminths infection status. (A) AnySTHs positive vs. Negative, (B) Ascaris positive vs Negative, (C) Trichuris positive vs. Negative, (D) Schistosoma positive vs Negative.

**Fig 8:**
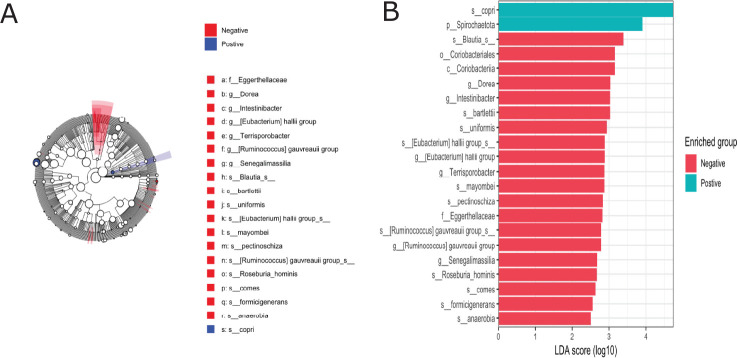
Taxonomic cladogram obtained from linear discriminant analysis effect size (LEfSe) highlights specific bacterial taxa that were relatively more abundant in AnySTHs Negative (red) or more abundant in AnySTHs positive (blue). Significant bacterial genera were determined by Kruskal-Wallis test (P, 0.05) with LDA score greater than or equal to 2.5.

**Fig 9:**
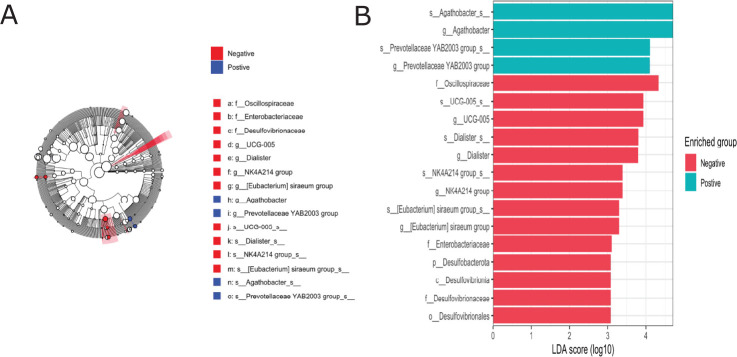
Taxonomic cladogram obtained from linear discriminant analysis effect size (LEfSe) highlights specific bacterial taxa that were relatively more abundant in Trichuris Negative (red) or more abundant in Trichuris positive (blue). Significant bacterial genera were determined by Kruskal-Wallis test (P < 0.05) with LDA score greater than or equal to 3.

Differences in relative abundance between STH-infected and non-infected groups were also assessed at lower taxonomic levels (Figs 5 and 7). The genera with the highest relative abundance were *Prevotella_9*, *Succinivibro*, *Faecalibacterium*, *Alloprevotella*, and *Agathobacter* in both children infected with any STHs and non-infected, but with no significant difference between the two groups **(Fig 5A).** Comparing the microbial taxa at the genus level by different helminth species **(Figs 5B–5D and 6A)** showed a significant increase in *Agathobacter* relative abundance among children infected with *Trichuris trichiura* compared to non-infected after adjusting for multiple comparisons correction (adjusted p = 0.001, **Fig 6A**). At the family level, a similar trend of high Lachnospiraceae (the family of *Agathobacter*) relative abundance was also observed in children infected with *Trichuris trichiura* compared to non-infected children (adjusted *p* = 0.002, **Figs 6B and 7).** When we applied linear discriminant analysis (LDA) effect size (LEfSe), an algorithm for discriminating high-dimensional biomarker of genomic features, to detect significant differences in bacterial taxonomies (LDA score > 2.5), we found 22 taxa with significant variation between the STH-negative and STH-positive groups **(Fig 8).** Using LEfSe to compare *Trichuris*-negative and positive groups, we found 18 taxa with significant differences in abundance **(Fig 9).**

The relationships between genus level microbial taxa with intensity of helminths infection (eggs per gram (epg)) as well as participant characteristics such as age, weight, and height were further investigated through Spearman’s correlation analysis (**S7 Fig)**. The most significant association was again with *Agathobacter*, which was significantly positively correlated with *Trichuris trichiura* egg counts (p < 0.05).

## Discussion

Our cross-sectional baseline 16S RNA data analysis provides insights into the characteristics of the gut microbiome profile among STH-infected versus non-infected Ethiopian school children. We found a nonsignificant decrease in trends of alpha diversity (Shannon index and chao1) among helminth-infected compared to uninfected subjects. However, children infected with *Trichuris trichiura* showed significantly lower microbial diversity. We also found that *Trichuris trichiura* infection correlated with broader compositional differences in the microbiome, although such differences were not observed with other helminths or for STH infection overall.

Most studies examining the association between helminths and microbial diversity have reported conflicting results [52,53]. A recent meta-analysis of 15 studies found a higher average Shannon index in helminth-positive individuals than those without helminth infection [52]. However, studies included in another meta-analysis reported either lower [16] or no significant difference [23,54] in average Shannon index among helminth-positive than helminth-negative subjects. Similar conflicting results were reported on the association between individual species of helminths and microbial diversity. Cupper et al. [16] reported a non-significant association between either *A*. *lumbricoides* or *T*. *trichiura* with alpha diversity among Ecuadorian Schoolchildren. Chen et al. [55] analyzed fecal samples from mothers and children and reported a significantly higher fecal microbiota alpha diversity in mothers but not in children infected with *T*. *trichiura* compared with noninfected. Tee et al. [56] also reported a significantly higher fecal microbiota alpha diversity among *T*. *trichiura* infected indigenous Malaysian population. In contrast, we found a significantly lower microbial diversity among children infected *T*. *trichiura*. We further examined beta diversity using a PCoA based on unweighted UniFrac distances and found broad compositional differences in the microbiome between study participants infected with *T*. *trichiura*, but no such differences for any other state of helminth infection tested. Others have reported no clear clustering of samples based on either overall helminth [23] or *T*. *trichiura* infection status [16]. The differences in the results reported by various studies on the link between helminth infection and microbial diversity may be due to variations in factors that impact microbiota composition. These factors can include the type of study population, environmental exposures, hygiene, diet, and antibiotic use, among other variables [57,58]. We, therefore, investigated the potential relationship between certain demographic and lifestyle factors (BCG vaccination, mode of delivery, history of diarrhea) and microbial diversity. We found no significant difference in alpha diversity. However, previous studies on travelers [59] and young children aged 0–3[60] have shown that diarrhea significantly changes the composition of gut bacteria. Comparing our findings with previous research is challenging due to differences in the study population and the complex causes of diarrhea [61]. Alternatively, the observed discrepancy among these studies could also be due to differences in study design, such as the timing of measurements and statistical methods used to examine microbial diversity.

Nevertheless, two conflicting hypotheses explain the impact of helminths on microbial alpha diversity. One hypothesis suggests that helminths suppress inflammation, leading to an increase in microbial alpha diversity [62–64]. Another hypothesis, based on mouse models of *T*. *muris* infection, suggests that repeated exposure to *T*. *muris* causes a decrease in microbial diversity early on during infection, which rapidly resolves over time [65]. Our data supports the latter hypothesis, as repeated exposure to STHs is common in endemic areas [66]. However, more controlled longitudinal studies are needed to confirm this hypothesis and explain the exact mechanism.

Our study revealed that Bacteroidetes, Firmicutes, and Proteobacteria were the most predominant taxa at the phylum level. This finding is consistent with previous studies conducted on rural African populations and is associated with consuming a diet rich in fiber and starch [67]. Although we did not analyze the detailed dietary patterns of the study population, previous studies in Ethiopia have reported that fiber and starch-based foods are commonly consumed [68,69]. When we compared participants with or without any STHs for an abundance of microbial taxa at the genus level, *Prevotella_9*, *Succinivibro*, *Faecalibacterium*, *Alloprevotella*, and *Agathobacter* were found to be abundant in both groups without significant differences between the two groups. Previous research conducted in various regions has shown a link between helminth infection and the abundance of different types of bacteria taxa. For example, Rosa et al. [21] found that the bacterial genera *Olsenella*, *Flavonifractor*, *Enterococcus*, and *Allobaculum* were significantly linked to STHs in Indonesia and Liberia. Similarly, Jenkins et al. [18] discovered that *Akkermansia* and *Lactococcus* were significantly more abundant in Helminth-infected individuals than those uninfected. It has been suggested that varying prevalence rates of different helminths and unique genetics and living conditions may explain the observation of distinct taxa of microbiome abundance in each study [53]. Nevertheless, in the context of Ethiopia, where STHs have been widely prevalent and endemic for decades [70,71], it is plausible that prior helminth infections may have already had an impact on the gut microbiota of both infected and uninfected individuals in the same way.

In this study, the significant increase in relative abundance of the *Agathobacter* genus (family Lachnospiraceae) found in *Trichuris trichiura* infected children is a novel observation and particularly intriguing. *Agathobacter* is an anaerobic, Gram-positive bacteria that produces butyrate in the human gut and has been linked to promoting gut health [72]. Butyrate is a short-chain fatty acid produced by anaerobic microorganisms through the fermentation of indigestible carbohydrates via the acetyl-coenzyme A (AcCoA) pathway [73]. It has been associated with preventing pathogen invasion, modulating the immune system, and slowing cancer progression [74]. According to an animal model, persistent *Trichuris muris* infection significantly increases the genus *Lactobacillus* [75]. This increase in *Lactobacilli* is believed to be a result of the immune-modulating effect of *Trichuris* infection, which could be part of a mutualistic relationship with the resident bacteria. [76] It is plausible that the increase in *Agathobactor* observed in this study is also a result of a similar mutualistic relationship with *Trichuris trichiura*. It is imperative to conduct further investigation on this aspect using well-defined and consistent experimental setups. Furthermore, we observed a positive correlation between the fecal egg count for *Trichuirs trichuria* and the abundance of *Agathobacter*. A study by Hayes et al. on the relationship between the establishment of *T*. *muris* and gut microbiota in a mouse model revealed that the eggs of T. muris require bacterial surface structures called fimbriae to hatch [77]. It is unclear whether a similar mechanism is necessary for *Trichuirs trichuria* or if *Agathobacter* is responsible for producing these structures. However, this could potentially explain the connection between egg abundance at that particular microbe.

Our findings should be interpreted considering the following limitations. Firstly, our cross-sectional analysis made it challenging to establish a causal relationship between STHs and the altered gut microbiome, such an analysis of causality requires a gut microbiome profile before the STH infection. Therefore, a longitudinal study is necessary to eliminate the possibility of reverse causation. We collected demographic and lifestyle information using self-reported questionnaires. However, this method is susceptible to misclassification and recall bias. Despite this, the questionnaire had been effective in a comparable population in Ethiopia in the past [78], increasing our findings’ validity. The school-based deworming program usually provides albendazole and mebendazole, while children may have also received other drugs like praziquantel from different providers. Although it is difficult to rule out this possibility completely, the study participants share similar socio-demographic backgrounds in accessing standard treatments. This suggests that any potential bias is nondifferential across study subjects and is unlikely to impact the study’s findings. Secondly, we did not assess the potential impact of sample freezing on the composition of microbiomes. Previous research has linked this practice to a decreased abundance of Bacteroides in fecal samples [79]. However, we used Norgen preservative before freezing our samples. This preservative has been tested as being highly effective at retaining accurate microbial composition both at freezing and room temperature, thereby minimizing bias due to freezing and transportation [80]. Third, the identification of STHs was conducted using standard microscopic examination, which may underestimate the true prevalence compared to highly sensitive PCR-based detection methods [81]. Fourth, the role of protozoan infection, such as *Entameba histolytica*, has been linked with changes in gut microbiome composition [82]. However, our study lacked data on protozoan infections and couldn’t examine the role protozoan infection plays in microbiome composition. Future studies are recommended to examine the possible role of protozoan infection on gut microbiota composition in the study area. Fifth, fecal microbiomes don’t necessarily reflect microbiome changes along the length of the entire gastrointestinal tract [83]. There may be differences in composition, for example, in the small intestine, that aren’t reflected in fecal composition changes.

Additionally, fecal samples more accurately reflect the composition in the lumen of the distal colon than the composition along the mucosal layer. Given the links between mucus composition and helminth infection [84], this could be an important area for future study. Finally, this study does not analyze the potential connections between helminths and microbial pathways.

Despite these limitations, to the best of our knowledge, this is the first study to examine the association of the gut microbiome with STH infection among young Ethiopian school children. Our findings showed a higher abundance of *Agathobactor* in children infected with *Trichuris trichiura*, suggesting the necessity for a mechanistic study to understand the underlying mechanisms.

## Supporting information

S1 Fig Rarefaction curves calculated for the number of amplicon sequence variants (ASVs) with increasing sequencing depth. Individual lines represent a sample, and N = 138. A) all samples; B) by sex (red = Female, blue = Male). The plateauing of the curves indicates that the majority of the species (ASVs) present in each sample have been detected at the sequencing depths obtained in this study.
(TIF)

S2 Fig Rarefaction curves calculated for the number of amplicon sequence variants (ASVs) by rarefying to 20,000 read sampling depth without replacement Individual lines represent a sample, and N = 138. A) all samples; B) by sex (red = Female, blue = Male). The plateauing of the curves indicates that the majority of the species (ASVs) present in each sample have been detected at the sequencing depths obtained in this study
(TIF)

S3 Fig Principal coordinate analysis (PCoA) based on weighted Bray Curtis matrix dissimilarity of overall fecal microbiota composition by different Helminths infection status. (A) AnySTHs Positive vs. Negative, (B) Ascaris Positive vs. Negative, (C) Trichuris Positive vs. Negative, (D) Schistosoma Positive vs. Negative. p-values resulted from PERMANOVA analysis between compared groups.
(TIF)

S4 Fig Ordination plots. Ordination plots derived from unconstrained Principal Components Analysis (PCA) based on the Aitchison distance, showing the overall composition of the microbial community at genus level. A) AnySTHs Positive vs. Negative, (B) Ascaris Positive vs. Negative, (C) Trichuris Positive vs. Negative, (D) Schistosoma Positive vs. Negative. Taxa that were present in less than five samples were excluded from this analysis. Data were transformed using center-log-ratio transformation. Names are given for taxa, which contributed most to overall microbial variation.
(TIF)

S5 Fig Heatmap of Spearman’s correlation analysis between the gut microbiome abundance (top 15 most abundant genera) with demographic factors (age, height, weight) and Helminth infection intensity (AL = *Ascaris lumbricoides*, HW *=* Hookworms, TT = *Trichuris trichiura)*. Significant correlations are marked by * *p* < 0.05; ** *p* < 0.01.
(TIF)

S6 Fig Microbiota diversity by selected demographic and lifestyle categories. Boxplots show alpha diversity (either Chao1 or Shannon diversity) as a function of BCG vaccination, Mode of delivery, deworming in the past six months, and episode of diarrhea in the past two weeks. A) BCG vaccination, Yes vs. No, (B) Mode of delivery Vaginal vs. C-section, (C) deworming in the past six months, yes vs. No, (D) episode of diarrhea in the past two weeks, Yes vs. No. Statistical significance was assessed with the Wilcoxon rank-sum
(TIF)

S7 Fig Principal coordinate analysis (PCoA) based on weighted Bray Curtis matrix dissimilarity of overall fecal microbiota composition by selected demographic and lifestyle categories A) BCG vaccination, Yes vs. No, (B) Mode of delivery Vaginal vs. C-section, (C) deworming in the past six months, yes vs. No, (D) episode of diarrhea in the past two weeks, Yes vs. No. P-values resulted from PERMANOVA analysis between compared groups.
(TIF)

S8 Fig Taxonomic cladogram obtained from linear discriminant analysis effect size (LEfSe) highlights specific bacterial taxa that were relatively more abundant in Ascaris Negative (red) or more abundant in Trichuris Positive (blue). Significant bacterial taxa were determined by Kruskal-Wallis test (P < 0.05) with log10 LDA score greater than or equal to 2.5.
(TIF)

S9 Fig Taxonomic cladogram obtained from linear discriminant analysis effect size (LEfSe) highlights specific bacterial taxa that were relatively more abundant in *Schistosoma* Negative (red) or more abundant in *Schistosoma* Positive (blue). Significant bacterial taxa were determined by Kruskal-Wallis test (P, 0.05) with log10 LDA score greater than or equal to 2.
(TIF)

S1 Table Sociodemographic characteristics and lifestyle factors, the original cohort. (PDF)

S1 File Data collection questionnaire. (DOCX)

S2 File Data. (RDS)

S1 Code R markdown codes used to analyze the data. (ZIP)
