# Supplementary figures and images for: Gut-microbiome profiles among Soil-transmitted helminths (STHs) infected Ethiopian children enrolled in the school-based mass deworming program
Source: PLoS Negl Trop Dis. 2024 Oct 15;18(10):e0012485. doi: 10.1371/journal.pntd.0012485 (PMC11478818; doi:10.1371/journal.pntd.0012485)

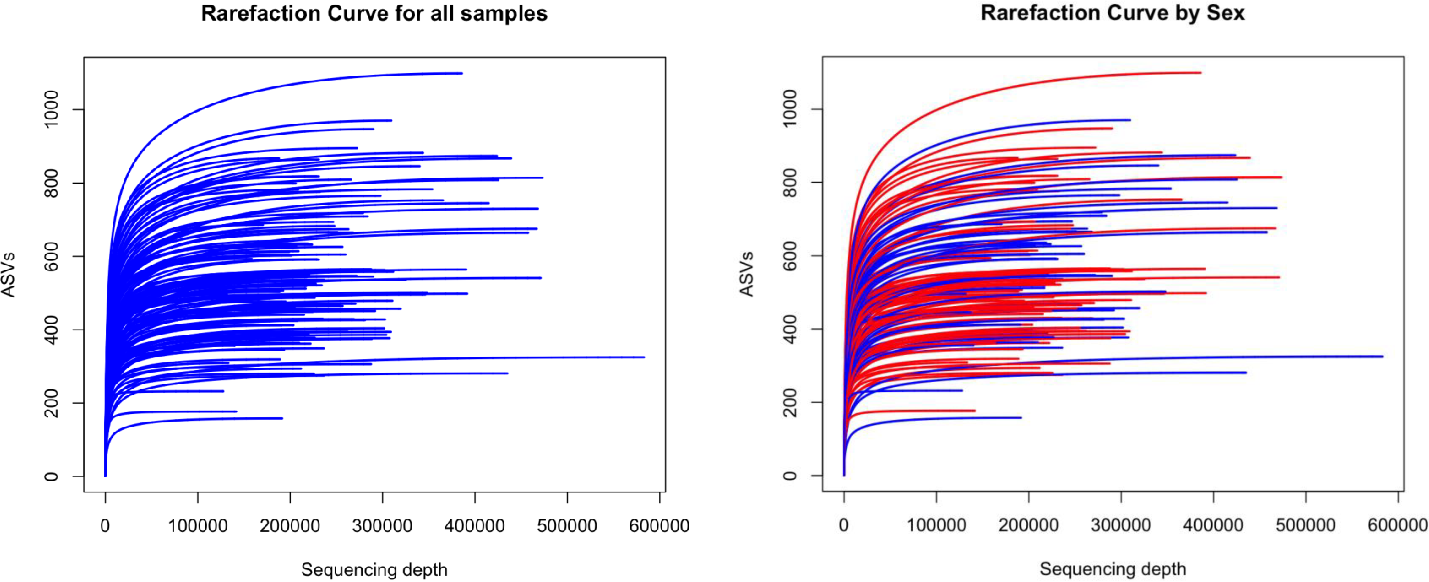

Supplement: S1 Fig — Individual lines represent a sample, and N = 138. A) all samples; B) by sex (red = Female, blue = Male). The plateauing of the curves indicates that the majority of the species (ASVs) present in each sample have been detected at the sequencing depths obtained in this study. (TIF) [file pntd.0012485.s001.tif]

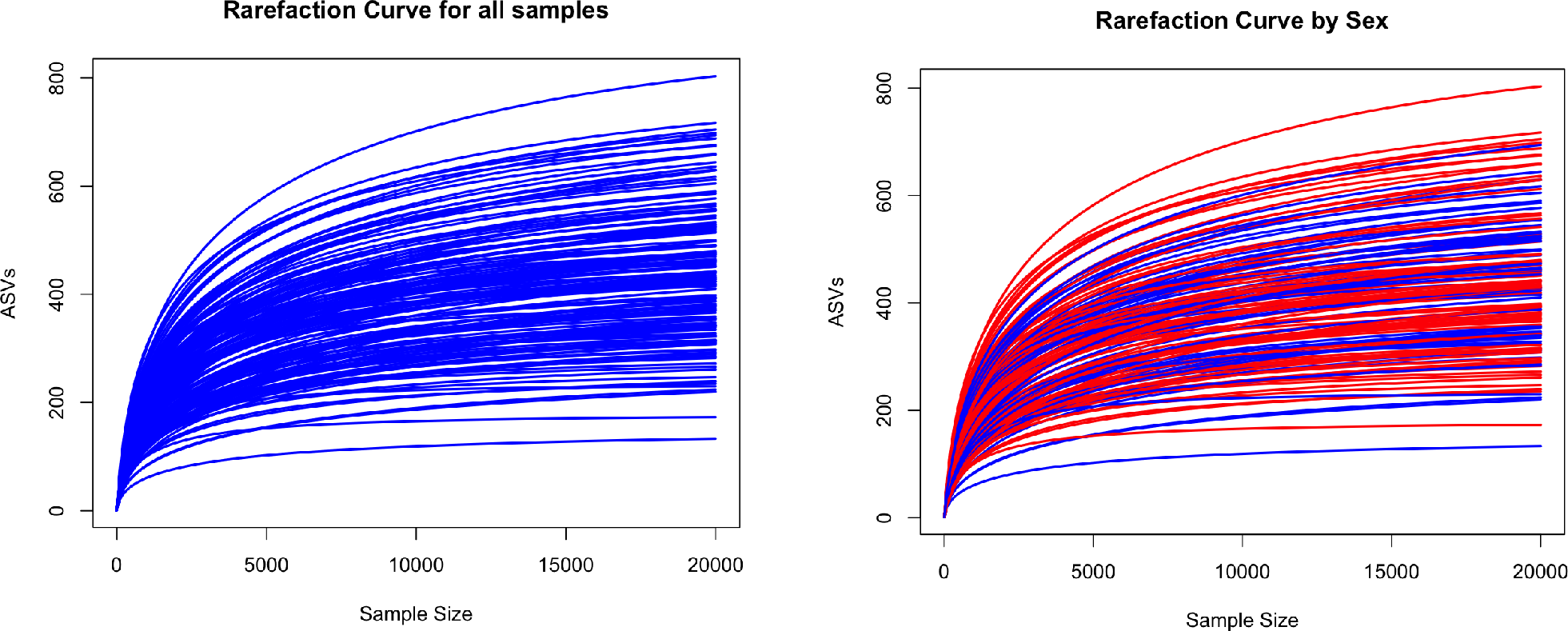

Supplement: S2 Fig — A) all samples; B) by sex (red = Female, blue = Male). The plateauing of the curves indicates that the majority of the species (ASVs) present in each sample have been detected at the sequencing depths obtained in this study (TIF) [file pntd.0012485.s002.tif]

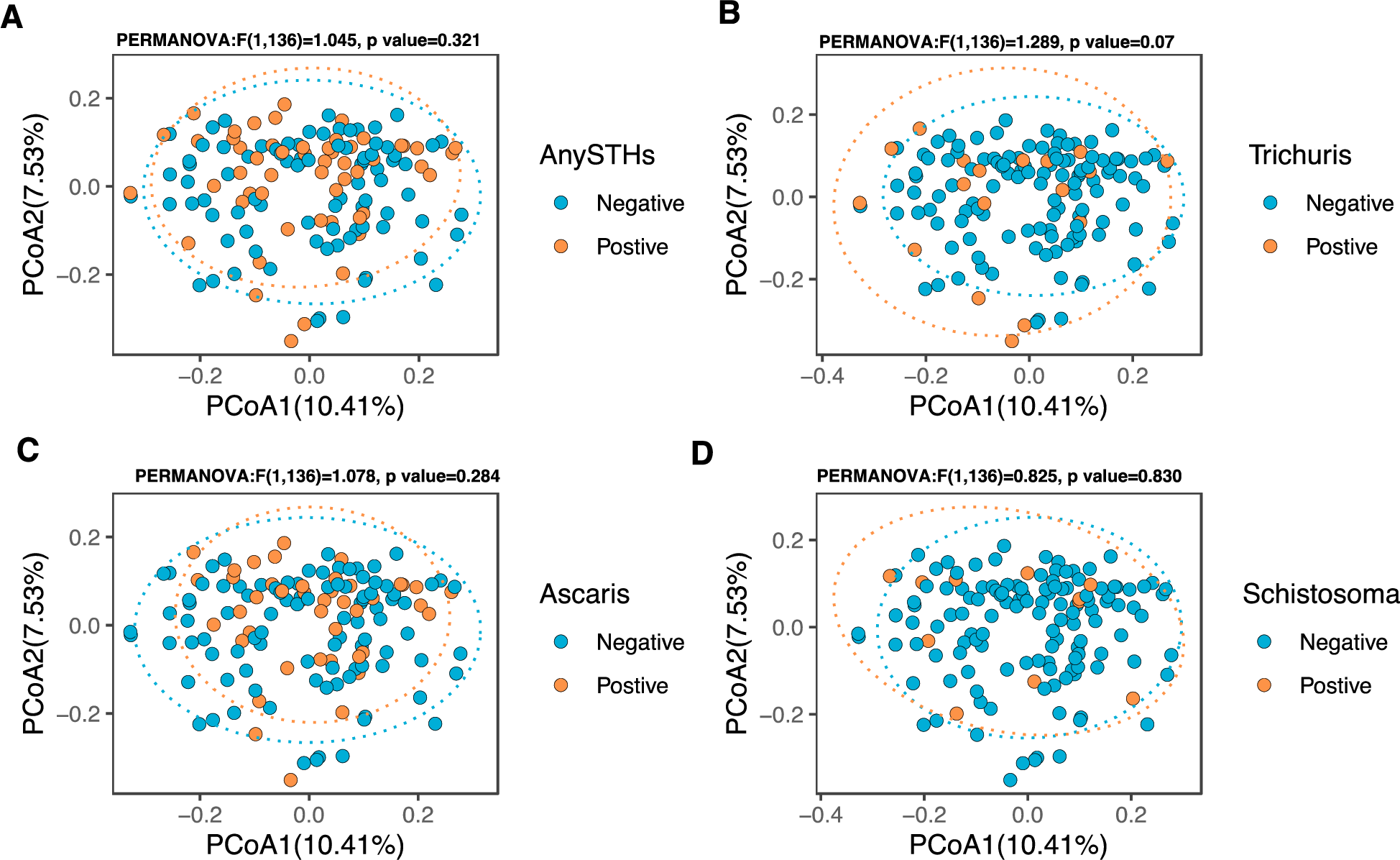

Supplement: S3 Fig — (A) AnySTHs Positive vs. Negative, (B) Ascaris Positive vs. Negative, (C) Trichuris Positive vs. Negative, (D) Schistosoma Positive vs. Negative. p-values resulted from PERMANOVA analysis between compared groups. (TIF) [file pntd.0012485.s003.tif]

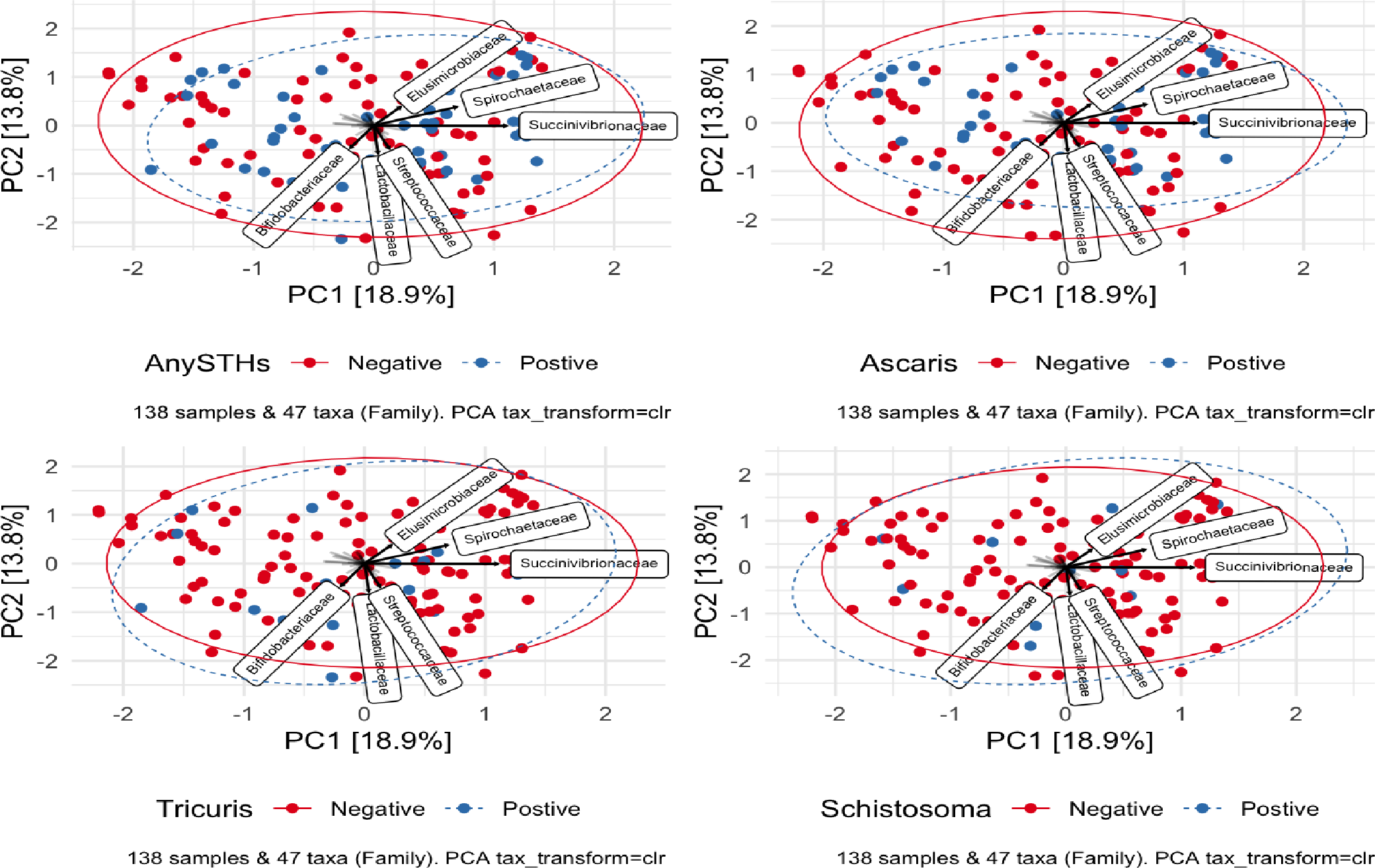

Supplement: S4 Fig — Ordination plots derived from unconstrained Principal Components Analysis (PCA) based on the Aitchison distance, showing the overall composition of the microbial community at genus level. A) AnySTHs Positive vs. Negative, (B) Ascaris Positive vs. Negative, (C) Trichuris Positive vs. Negative, (D) Schistosoma Positive vs. Negative. Taxa that were present in less than five samples were excluded from this analysis. Data were transformed using center-log-ratio transformation. Names are given for taxa, which contributed most to overall microbial variation. (TIF) [file pntd.0012485.s004.tif]

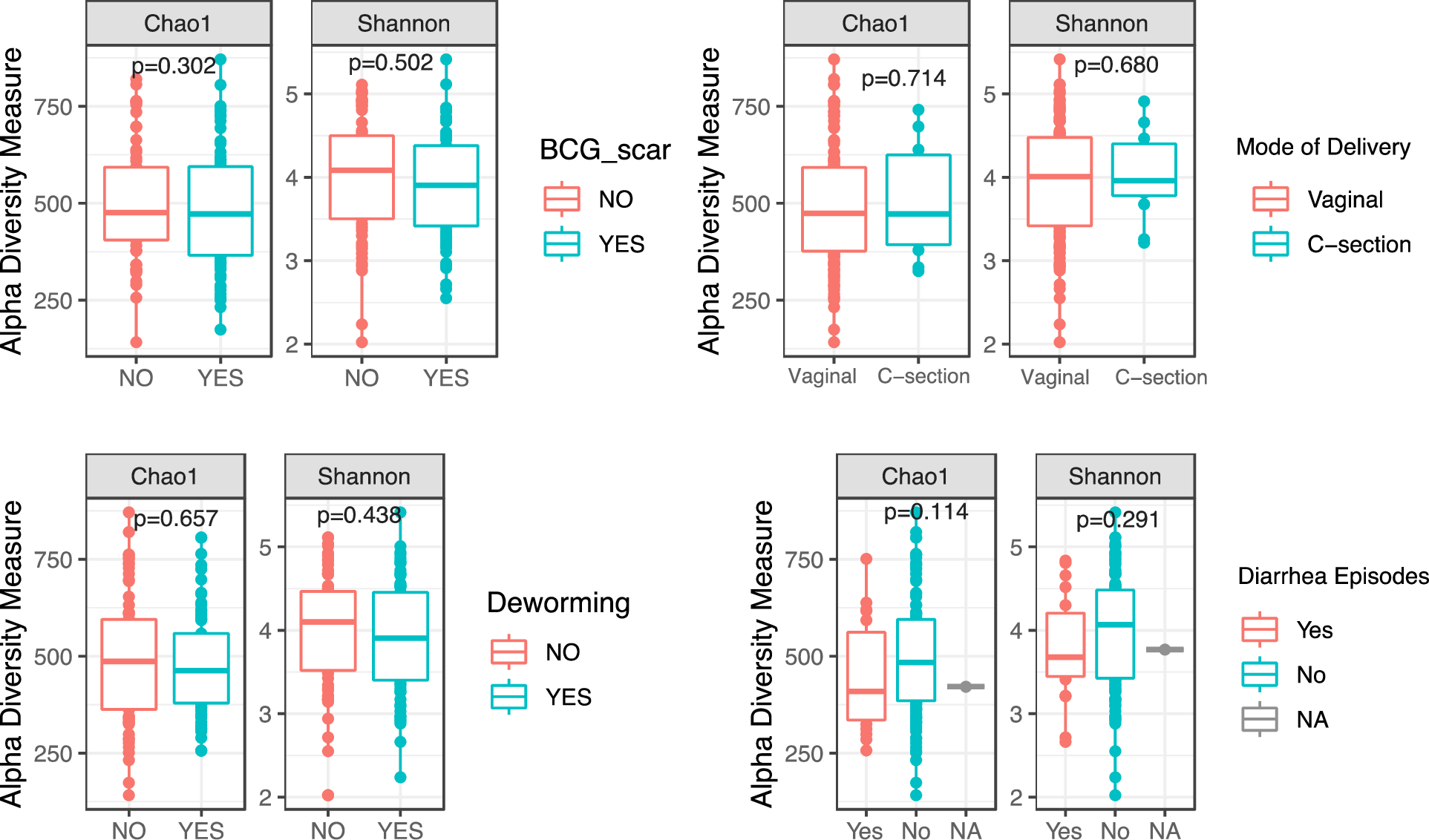

Supplement: S5 Fig — Significant correlations are marked by * p < 0.05; ** p < 0.01. (TIF) [file pntd.0012485.s005.tif]

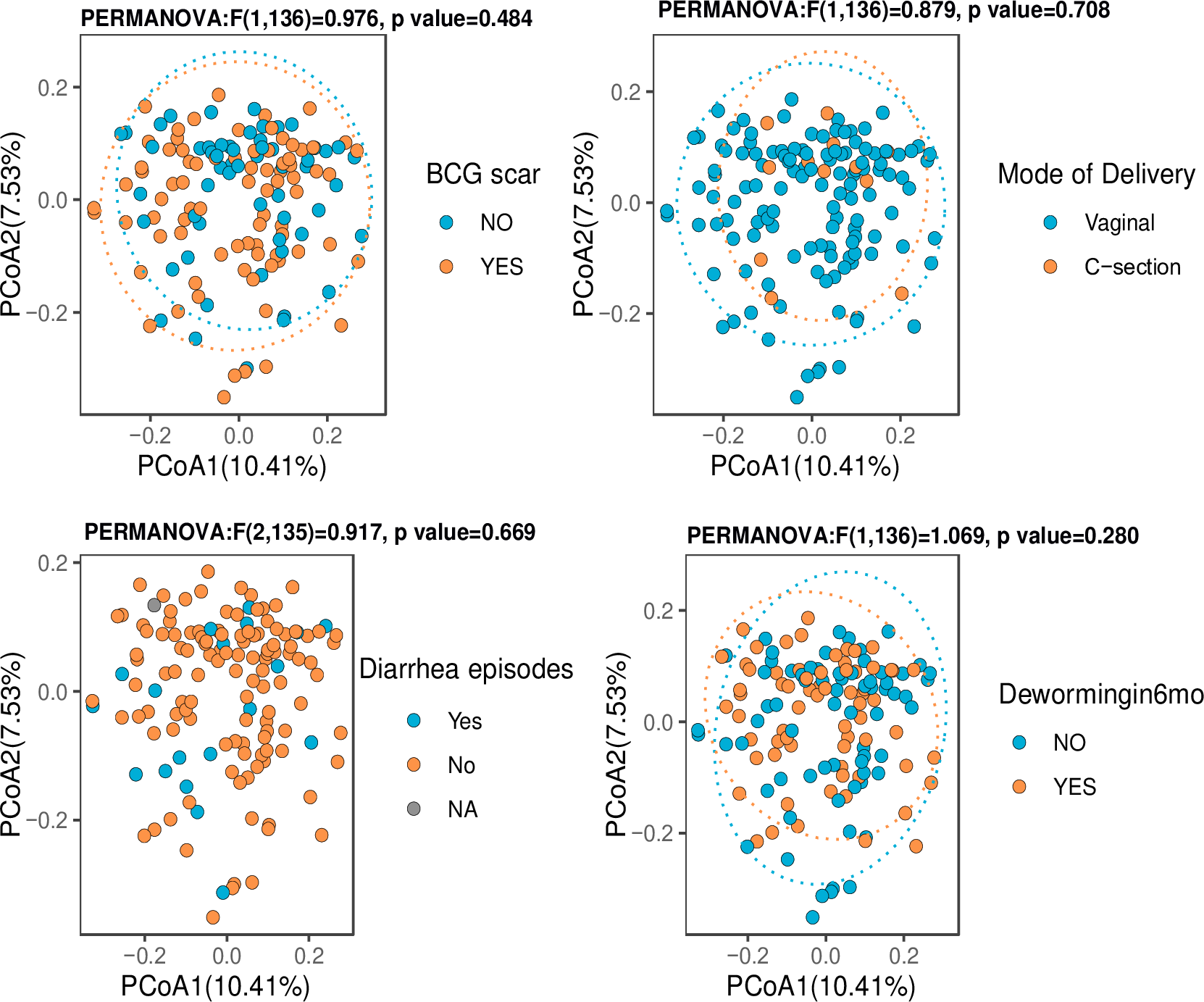

Supplement: S6 Fig — Boxplots show alpha diversity (either Chao1 or Shannon diversity) as a function of BCG vaccination, Mode of delivery, deworming in the past six months, and episode of diarrhea in the past two weeks. A) BCG vaccination, Yes vs. No, (B) Mode of delivery Vaginal vs. C-section, (C) deworming in the past six months, yes vs. No, (D) episode of diarrhea in the past two weeks, Yes vs. No. Statistical significance was assessed with the Wilcoxon rank-sum (TIF) [file pntd.0012485.s006.tif]

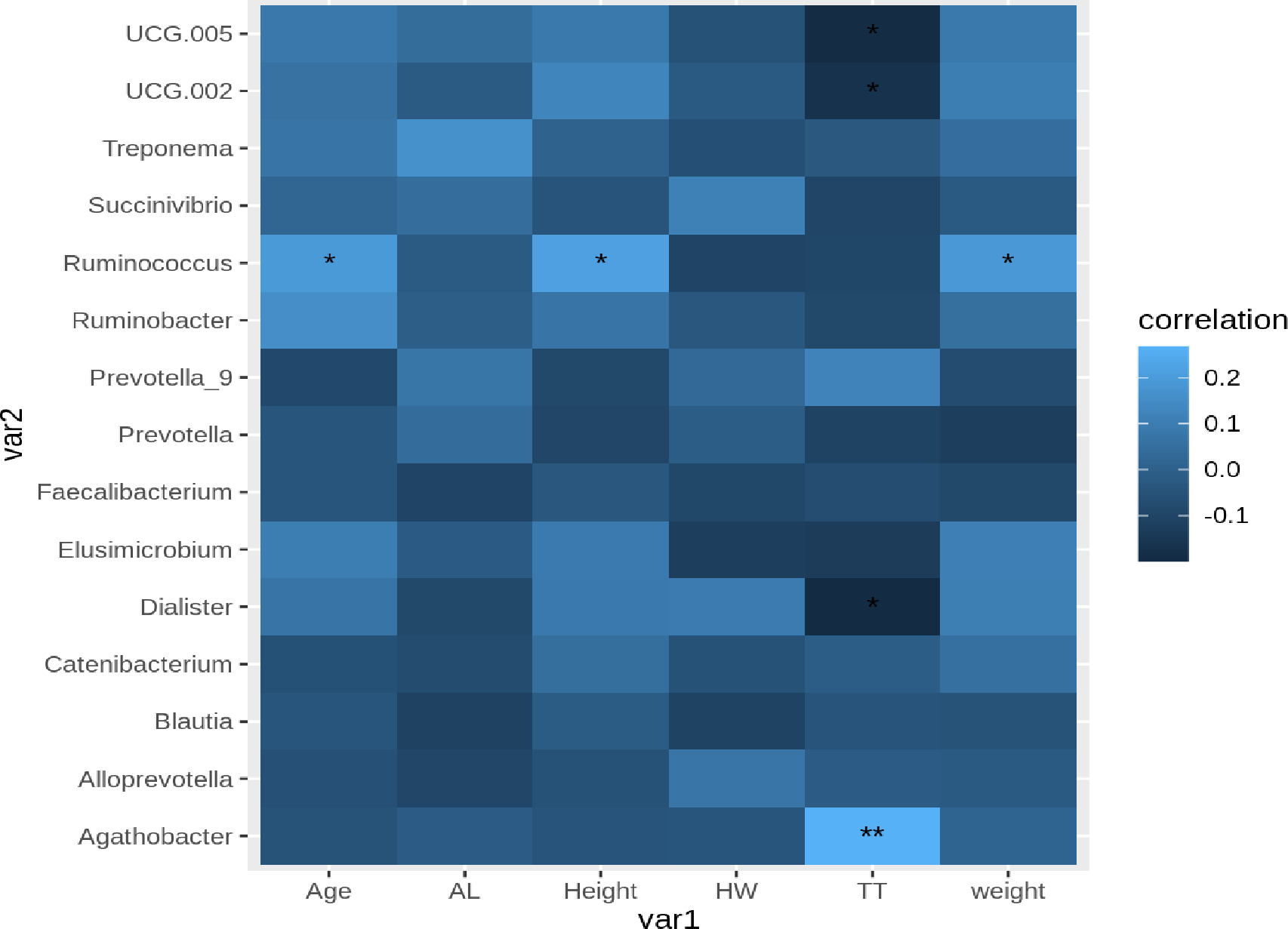

Supplement: S7 Fig — Principal coordinate analysis (PCoA) based on weighted Bray Curtis matrix dissimilarity of overall fecal microbiota composition by selected demographic and lifestyle categories A) BCG vaccination, Yes vs. No, (B) Mode of delivery Vaginal vs. C-section, (C) deworming in the past six months, yes vs. No, (D) episode of diarrhea in the past two weeks, Yes vs. No. P-values resulted from PERMANOVA analysis between compared groups. (TIF) [file pntd.0012485.s007.tif]

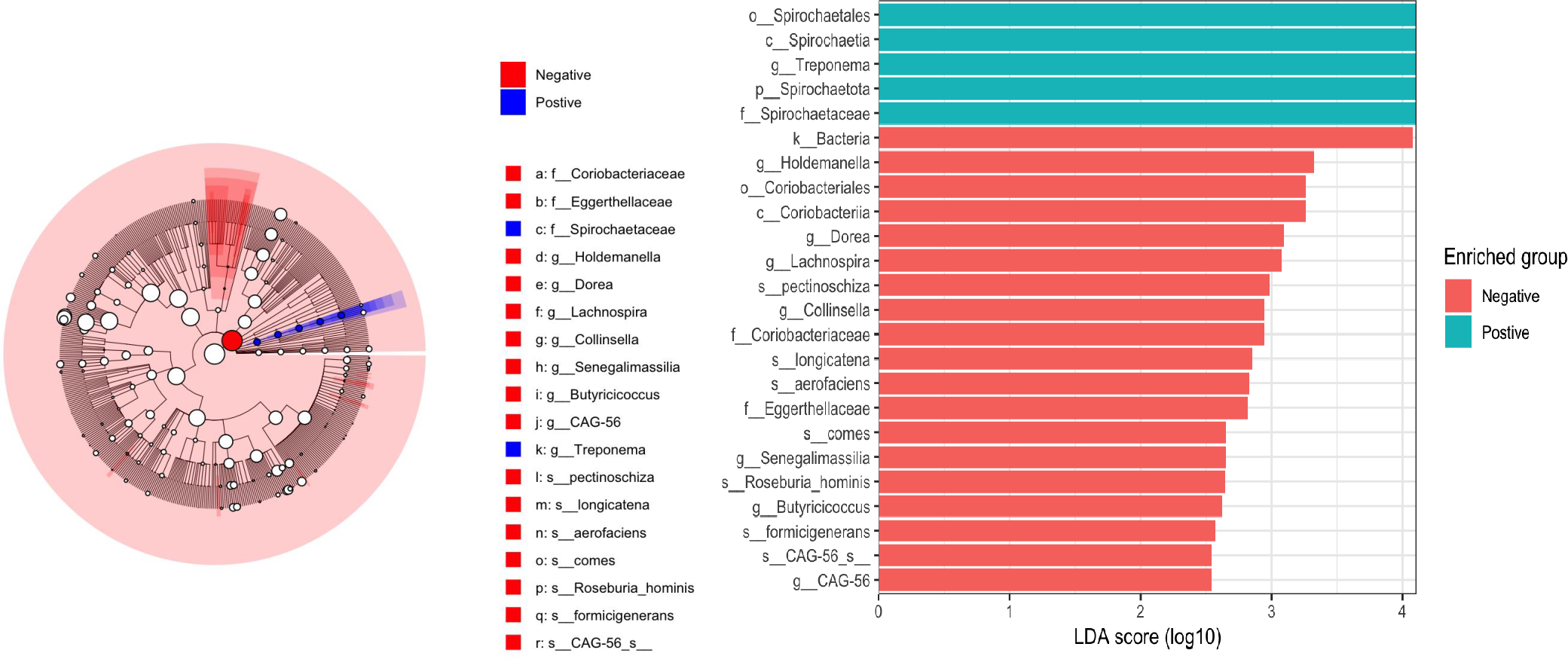

Supplement: S8 Fig — Taxonomic cladogram obtained from linear discriminant analysis effect size (LEfSe) highlights specific bacterial taxa that were relatively more abundant in Ascaris Negative (red) or more abundant in Trichuris Positive (blue). Significant bacterial taxa were determined by Kruskal-Wallis test (P < 0.05) with log10 LDA score greater than or equal to 2.5. (TIF) [file pntd.0012485.s008.tif]

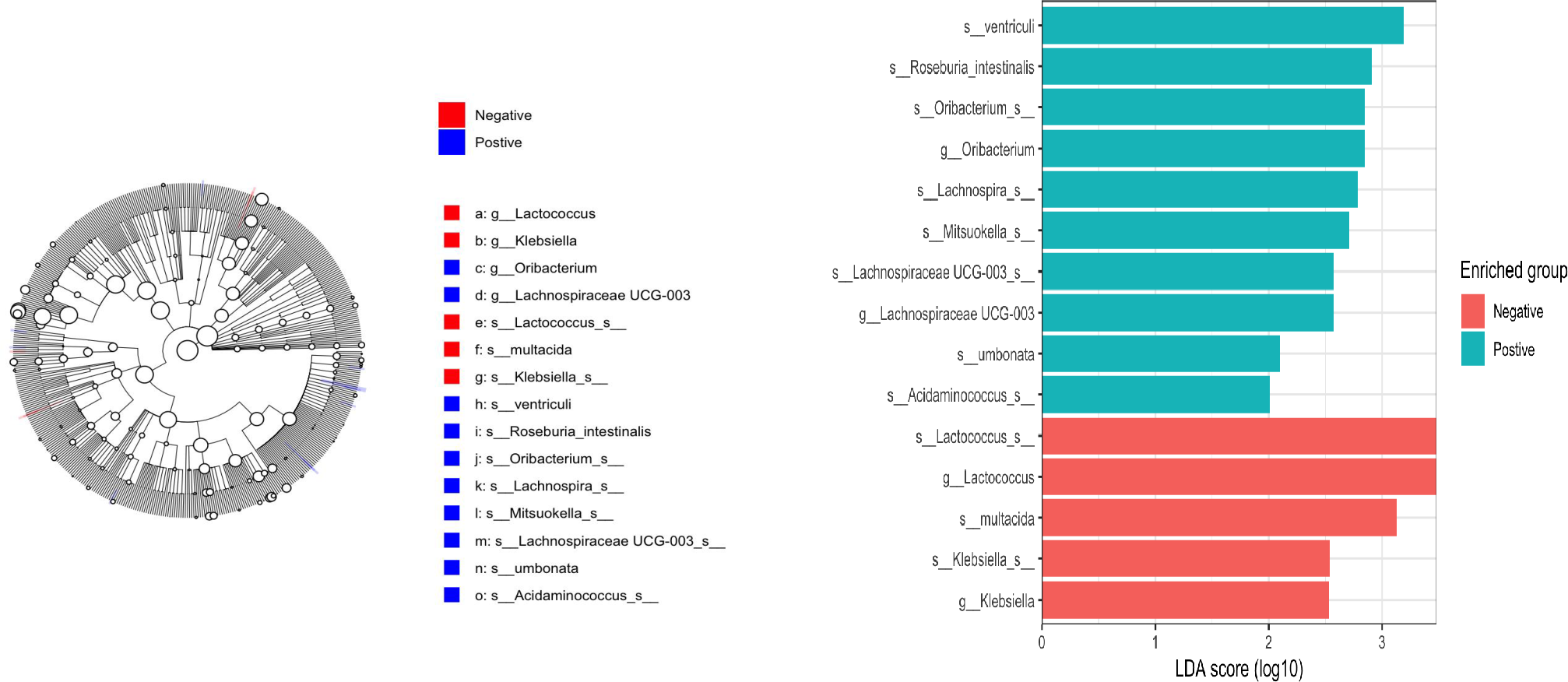

Supplement: S9 Fig — Taxonomic cladogram obtained from linear discriminant analysis effect size (LEfSe) highlights specific bacterial taxa that were relatively more abundant in Schistosoma Negative (red) or more abundant in Schistosoma Positive (blue). Significant bacterial taxa were determined by Kruskal-Wallis test (P, 0.05) with log10 LDA score greater than or equal to 2. (TIF) [file pntd.0012485.s009.tif]
