# Supplementary material for: Gut-microbiome profiles among Soil-transmitted helminths (STHs) infected Ethiopian children enrolled in the school-based mass deworming program
Source: PLoS Negl Trop Dis. 2024 Oct 15;18(10):e0012485. doi: 10.1371/journal.pntd.0012485 (PMC11478818; doi:10.1371/journal.pntd.0012485)
Supplement: S1 Table — (PDF) [file pntd.0012485.s010.pdf]

S1 Table. Sociodemographic characteristics and lifestyle factors, the original cohort

Variable	Frequency (%)
**Sex**	
Male	498 (48.1%)
Female	538 (51.9%)
**Age Group**	
5 - 9	425 (41%)
10 - 14	544 (52.5%)
> 14	67 (6.5%)
**Mode of Child Delivery**	
Vaginal	950 (91.7%)
C-Section	75 (2.2%)
Unknown	11 (1.1%)
**Family Size**	
1 - 3	63 (6.1%)
4 - 5	432 (41.7%)
> 5	540 (52.2%)
**Vaccination History**	
Not Vaccinated	724 (29%)
Vaccinated	300 (70%)
Unknown	10 (1%)
**Presence of BCG Scar**	
No	445 (43%)
Yes	590 (56.9%)
**Deworming Pill History**	
No	390 (37.6%)
Yes	646 (62.4%)
**Residence Type**	
Urban	1021 (98.7%)
Rural	13 (1.3%)
