# Supplementary material for: Gut-microbiome profiles among Soil-transmitted helminths (STHs) infected Ethiopian children enrolled in the school-based mass deworming program
Source: PLoS Negl Trop Dis. 2024 Oct 15;18(10):e0012485. doi: 10.1371/journal.pntd.0012485 (PMC11478818; doi:10.1371/journal.pntd.0012485)
Supplement: S1 File — (DOCX) [file pntd.0012485.s011.docx]

**School, Child, and Interviewer Identification**

| School name  _____________________  Name of Teacher/Class  ______________________ | Name of Child:  _________________________________  Unique Id number   \|  \| \| --- \| | Name of Interviewer  _____________________  Date of Interview (dd/mm/aaaa)   \| **/ /** \| \| --- \| |
| --- | --- | --- | --- | --- |

**Eligibility requirements**

| **A** | Has the parent consented? | \| **⃞** \| Yes \| *Go to question B* \| **⃞** \| No \| *Stop interview* \| \| --- \| --- \| --- \| --- \| --- \| --- \| |
| --- | --- | --- | --- | --- | --- | --- | --- | --- |
| **B** | Has the child assented? | \| **⃞** \| Yes \| *Go to question 1* \| **⃞** \| No \| *Stop interview* \| \| --- \| --- \| --- \| --- \| --- \| --- \| |

**Measures taken by the interviewer**

| **1** | Indicate if child attends morning, afternoon, or evening class | \| **⃞** \| Morning \| (1) \| **⃞** \| Afternoon \| (2) \| **⃞** \| Evening \| (3) \| \| --- \| --- \| --- \| --- \| --- \| --- \| --- \| --- \| --- \| |
| --- | --- | --- | --- | --- | --- | --- | --- | --- | --- | --- | --- |
| **2** | What is the sex of the child? | \| **⃞** \| Girl \| (1) \|  \| **⃞** \| Boy \| (0) \| \| --- \| --- \| --- \| --- \| --- \| --- \| --- \| |
| **3** | When is the child’s birthday? (dd/mm/aaaa) | \| **/ /** \| Age \|  \| *Don’t know* \| **⃞** \| \| --- \| --- \| --- \| --- \| --- \| |
| **4** | Weight of the child? | \|  \|  \| Kg \|  \| *(Not measured)* \| **⃞** \| \| --- \| --- \| --- \| --- \| --- \| --- \| |
| **5** | Height of the child? | \|  \|  \| Meters \|  \| *(Not measured)* \| **⃞** \| \| --- \| --- \| --- \| --- \| --- \| --- \| |
| **6** | Are the child’s fingernails dirty? | \| **⃞** \| Yes \| (1) \| **⃞** \| No \| (0) \| **⃞** \| *Don’t know* \| (99) \| \| --- \| --- \| --- \| --- \| --- \| --- \| --- \| --- \| --- \| |

**Level of Knowledge on Soil-transmitted helminths**

| **i** | **A-** Do you know how intestinal worms / parasites are transmitted? | | | | \| **⃞** \| Yes \| (1) \|  \| **⃞** \| No \| (0) \| \| --- \| --- \| --- \| --- \| --- \| --- \| --- \| | |
| --- | --- | --- | --- | --- | --- | --- | --- | --- | --- | --- | --- | --- | --- |
|  | **B-** If yes, how?   \| **⃞** *Not applicable (NA)* \| \| --- \| | \| 1) \|  \| \| --- \| --- \| \| 2) \|  \| \| 3) \|  \| \| 4) \|  \| | | | | |
| **II** | **A**- Do you know why worms / parasites are bad for your health? | | | | \| **⃞** \| Yes \| (1) \|  \| **⃞** \| No \| (0) \| \| --- \| --- \| --- \| --- \| --- \| --- \| --- \| | |
|  | **B-** If yes, why are they bad?   \| **⃞** *Not applicable (NA)* \| \| --- \| | | | \| 1) \|  \| \| --- \| --- \| \| 2) \|  \| \| 3) \|  \| \| 4) \|  \| | | |
| **III** | **A**- Do you know how you can avoid getting these worms / parasite infection? | | | | | \| **⃞** \| Yes \| (1) \|  \| **⃞** \| No \| (0) \| \| --- \| --- \| --- \| --- \| --- \| --- \| --- \| |
|  | **B-** If yes, how?   \| **⃞** *Not applicable (NA)* \| \| --- \| | | \| 1) \|  \| \| --- \| --- \| \| 2) \|  \| \| 3) \|  \| \| 4) \|  \| | | | |

**Questionnaire on risk factors**

| **7** | | **A-** Where do you live?  **B-** Where do you live (street, passage, sector, block, lot)? | | \| Community: ............................................... \| **⃞** *Don’t know* (99) \| \| --- \| --- \| \| Address: .................................................  .................................................................... \| **⃞** *Don’t know* (99)  **⃞** *Not applicable* (NA) \| | |
| --- | --- | --- | --- | --- | --- | --- | --- | --- | --- |
| **8** | | Is your house made out of noble or rustic materials? | | \| **⃞** \| Noble \| (1) \| **⃞** \| Rustic \| (0) \| **⃞** \| *Don’t know* \| (99) \| \| --- \| --- \| --- \| --- \| --- \| --- \| --- \| --- \| --- \| | |
| **9** | | In your house, do you Cook with gas, kerosene, coal, o wood? | | \| **⃞** \| Gas \| (0) \| **⃞** \| Coal \| (2) \| **⃞** \| Others____ \| (4) \| \| --- \| --- \| --- \| --- \| --- \| --- \| --- \| --- \| --- \| \| **⃞** \| Kerosene \| (1) \| **⃞** \| Wood \| (3) \| **⃞** \| *Don’t know* \| *(99)* \| | |
| **10** | | Do you have electricity at your house? | | \| **⃞** \| Yes \| (1) \| **⃞** \| No \| (0) \| **⃞** \| *Don’t know* \| (99) \| \| --- \| --- \| --- \| --- \| --- \| --- \| --- \| --- \| --- \| | |
| **11** | | Does your family own a radio? | | \| **⃞** \| Yes \| (1) \| **⃞** \| No \| (0) \| **⃞** \| *Don’t know* \| (99) \| \| --- \| --- \| --- \| --- \| --- \| --- \| --- \| --- \| --- \| | |
| **12** | | Does your family own a television? | | \| **⃞** \| Yes \| (1) \| **⃞** \| No \| (0) \| **⃞** \| *Don’t know* \| (99) \| \| --- \| --- \| --- \| --- \| --- \| --- \| --- \| --- \| --- \| | |
| **13** | | **A-** Do you have potable water in your house? | | \| **⃞** \| Yes \| (1) \| **⃞** \| No \| (0) \| **⃞** \| *Don’t know* \| (99) \| \| --- \| --- \| --- \| --- \| --- \| --- \| --- \| --- \| --- \| | |
|  |  | **B-** If not, where do you get your water from?   \| **⃞** *Not applicable (NA)* \| \| --- \| | | \| **⃞** \| Neighbour \| (0) \|  \| **⃞** \| Tank \| (4) \| \| --- \| --- \| --- \| --- \| --- \| --- \| --- \| \| **⃞** \| River \| (1) \|  \| **⃞** \| Public fountain \| (5) \| \| **⃞** \| Well \| (2) \|  \| **⃞** \| Others___________ \| (6) \| \| **⃞** \| Truck \| (3) \|  \| **⃞** \| *Don’t know* \| (99) \| | |
| **14** | | In your house do you drink your water directly or do you treat it (boiling or bleaching)? | | \| **⃞** \| Directly \| (1) \| **⃞** \| Treated \| (0) \| **⃞** \| *Don’t know* \| (99) \| \| --- \| --- \| --- \| --- \| --- \| --- \| --- \| --- \| --- \| | |
| **15** | | At home, is your latrine inside or outside the house? | | \| **⃞** \| Inside \| (1) \| **⃞** \| Outside \| (0) \| **⃞** \| *Don’t know* \| (99) \| \| --- \| --- \| --- \| --- \| --- \| --- \| --- \| --- \| --- \| | |
| **16** | | Is your latrine connected with the sewage system, a ditch, the river, or a well? | | \| **⃞** \| Sewage \| (0) \| **⃞** \| River \| (2) \| \| --- \| --- \| --- \| --- \| --- \| --- \| \| **⃞** \| Ditch \| (1) \| **⃞** \| Well \| (3) \| \| **⃞** *Don’t know* (99) \| \| \| \| \| \| | |
| **17** | | Do you bath in the Itaya river – always, sometimes, or never? | | \| **⃞** \| Always \| (0) \| **⃞** \| Never \| (2) \| \| --- \| --- \| --- \| --- \| --- \| --- \| \| **⃞** \| Sometimes \| (1) \| **⃞** \| *Don’t know* \| (99) \| | |
| **18** | | Do you defecate (#2) in the open air – always, sometimes, or never? | | \| **⃞** \| Always \| (0) \| **⃞** \| Never \| (2) \| \| --- \| --- \| --- \| --- \| --- \| --- \| \| **⃞** \| Sometimes \| (1) \| **⃞** \| *Don’t know* \| (99) \| | |
| **19** | | Do you use toilet paper to wipe your bum after you have defecated (#2) – always, sometimes, or never? | | \| **⃞** \| Always \| (0) \| **⃞** \| Never \| (2) \| \| --- \| --- \| --- \| --- \| --- \| --- \| \| **⃞** \| Sometimes \| (1) \| **⃞** \| *Don’t know* \| (99) \| | |
| **20** | | **A-** Do you wash your hands after going to the bathroom – always, sometimes, or never?  **B-** How do you wash your hands after going to the bathroom – with water only or with soap and water?   \| **⃞** *Not applicable (NA)* \| \| --- \|   **C-** If with soap and water, do you use soap always, sometimes, or never?   \| **⃞** *Not applicable (NA)* \| \| --- \| | | \| **⃞** \| Always \| (0) \| **⃞** \| Never \| (2) \| \| --- \| --- \| --- \| --- \| --- \| --- \| \| **⃞** \| Sometimes \| (1) \| **⃞** \| *Don’t know* \| (99) \|  \| **⃞** \| Water \| (0) \| **⃞** \| Soap and water \| (1) \| **⃞** \| *Don’t know* \| (99) \| \| --- \| --- \| --- \| --- \| --- \| --- \| --- \| --- \| --- \|  \| **⃞** \| Always \| (0) \| **⃞** \| Never \| (2) \| \| --- \| --- \| --- \| --- \| --- \| --- \| \| **⃞** \| Sometimes \| (1) \| **⃞** \| *Don’t know* \| (99) \| | |
| **21** | | **A-** Do you wash your hands before eating – always, sometimes, or never?  **B-** How do you wash your hands before eating – with water only or with soap and water?   \| **⃞** *Not applicable (NA)* \| \| --- \|   **C-** ¿ If with soap and water, do you use soap always, sometimes, or never?   \| **⃞***Not applicable (NA)* \| \| --- \| | | \| **⃞** \| Always \| (0) \| **⃞** \| Never \| (2) \| \| --- \| --- \| --- \| --- \| --- \| --- \| \| **⃞** \| Sometimes \| (1) \| **⃞** \| *Don’t know* \| (99) \|  \| **⃞** \| Water \| (0) \| **⃞** \| Soap and water \| (1) \| **⃞** \| *Don’t know* \| (99) \| \| --- \| --- \| --- \| --- \| --- \| --- \| --- \| --- \| --- \|  \| **⃞** \| Always \| (0) \| **⃞** \| Never \| (2) \| \| --- \| --- \| --- \| --- \| --- \| --- \| \| **⃞** \| Sometimes \| (1) \| **⃞** \| *Don’t know* \| (99) \| | |
| **22** | | **A-** What is your favourite fruit that you eat?  **B-** Do you wash your fruits before eating - always, sometimes, or never? | | Fruit: ………………………………….   \| **⃞** \| Never \| (0) \| **⃞** \| Always \| (2) \| \| --- \| --- \| --- \| --- \| --- \| --- \| \| **⃞** \| Sometimes \| (1) \| **⃞** \| *Don’t know* \| (99) \| | |
| **26** | | Do you walk barefoot - always, sometimes, or never? | | \| **⃞** \| Never \| (0) \| **⃞** \| Always \| (2) \| \| --- \| --- \| --- \| --- \| --- \| --- \| \| **⃞** \| Sometimes \| (1) \| **⃞** \| *Don’t know* \| (99) \| | |
| **27** | | When you are at home do you prefer to use sandals or shoes? | | \| **⃞** \| Does not use any \| (0) \| **⃞** \| Shoes \| (2) \| \| --- \| --- \| --- \| --- \| --- \| --- \| \| **⃞** \| Sandals \| (1) \| **⃞** \| *Don’t know* \| (99) \| | |
| **28** | | In which activities of the day are you barefoot?   \| **⃞** *Not applicable (NA)* \| \| --- \| | | A- Activity: ………..………………………………….  B- Activity: ………..………………………………….  C- Activity: ………..…………………………………. | |
| **29** | | **A-** Did your parents or your professor gave you a deworming pill?  **B-** If yes, when was the last time they gave you such deworming pill? | | \| **⃞** \| Yes \| (1) \| **⃞** \| No \| (0) \| **⃞** \| *Don’t know* \| (99) \| \| --- \| --- \| --- \| --- \| --- \| --- \| --- \| --- \| --- \|  \|  \| Months \| **⃞** \| More than a year (13) \| **⃞**  **⃞** \| *Don’t know (99)*  Not applicable (NA) \| \| --- \| --- \| --- \| --- \| --- \| --- \| | |
| **30** | | **A-** How many people live in your house? Enumerate them…  **B-** How many children younger than 12 years old live in your house? | | \|  \| People  (including you) \| **⃞** \| *Don’t know (99)* \| \| --- \| --- \| --- \| --- \| \|  \| Children \| **⃞** \| *Don’t know (99)* \| | |
|  | **Comments:** | |  | |  |
|  |  | | | |  |
|  |  | | | |  |
